# New Methodologies and Techniques for Biomonitoring Pesticide Exposure in Agricultural Workers: A Systematic Review

**DOI:** 10.3390/toxics13020104

**Published:** 2025-01-28

**Authors:** Andreia Moreira, Joana Guedes, Manuela Vieira da Silva

**Affiliations:** 1 Doctoral Programme in Occupational Safety and Health Student (DemSSO), Faculty of Engineering of University of Porto, Rua Dr. Roberto Frias, s/n, 4200-465 Porto, Portugal; add@ess.ipp.pt; 2 REQUIMTE/LAQV, E2S, Polythecnic of Porto, Rua Dr. António Bernardino de Almeida no. 400, 4200-072 Porto, Portugal; 3 Associated Laboratory for Energy, Transports and Aeronautics (PROA/LAETA), Faculty of Engineering of University of Porto, Rua Dr. Roberto Frias, s/n, 4200-465 Porto, Portugal; jccg@fe.up.pt; 4 EPIUnit—Institute of Public Health, University of Porto, Rua das Taipas no. 135, 4050-600 Porto, Portugal

**Keywords:** new techniques, biomonitoring, pesticide exposure, agricultural workers

## Abstract

Biomonitoring allows for the assessment of internal exposure to various pesticides and metabolites. Following PRISMA guidelines, this systematic review aims to summarise innovative biomonitoring techniques for assessing pesticide exposure in agricultural workers, their advantages and limitations, and their applicability. The search of the Medline/PubMed, ScienceDirect, Scopus, and Web of Science databases identified 14 articles dealing with new techniques for biomonitoring pesticide exposure in agricultural workers. These new methodologies have identified several biomarkers associated with exposure to organophosphates. Most of the included studies combine and/or improve traditional methods to overcome their limitations. This leads to more sensitive, specific, and precise techniques with lower detection and quantification limits. Therefore, it is necessary to thoroughly analyse and test new biomonitoring methods for assessing pesticide exposure. These techniques can complement qualitative risk assessments and aid in developing strategies to protect public health and the environment.

## 1. Introduction

Pesticides are continuously being used, becoming a permanent risk to humans and the environment. Exposure to these chemical substances, many of which are known to be toxic to those who encounter them, may occur via different routes of exposure, such as dermal contact, inhalation, and ingestion [1,2,3].

Several studies point to the importance of assessing exposure to these products among agricultural workers who handle or are in contact with pesticides in the course of their professional work, given their higher exposure—due to greater use in this sector—and the lack of training and adoption of safe practices in handling pesticides, as mentioned by many others [3,4,5,6,7].

In this sense, biomonitoring often complements more qualitative risk assessments based on data self-reported by workers in questionnaires and interviews [8,9,10,11,12]. Biomonitoring allows for the assessment of internal exposure to different pesticides and metabolites and is carried out by identifying and quantifying different known biomarkers for the different types of active substances [13,14].

Biomonitoring offers several advantages over other methods of assessing pesticide exposure: it provides a direct measure of the internal dose of pesticides in the body, reflecting the actual exposure and potential health risks to individuals [8,15]. It allows for the assessment of biomarkers that may indicate early biological effects and potential toxicity, providing insight into the adverse effects of pesticide exposure at a biochemical level [13,14]. This methodology can help identify periods of high pesticide exposure and assess the effectiveness of protective measures, allowing targeted interventions to minimise health risks [8].

Biomarkers can be divided into three main categories, exposure, effect, and susceptibility, each of which plays a different role in assessing the impact of chemical agents on the body and should be considered in this context. Exposure biomarkers assess the presence of an exogenous chemical substance or product resulting from the interaction between xenobiotics and target molecules or cells in biological samples. In turn, susceptibility biomarkers indicate the predisposition of an individual organism to respond to exposure to a xenobiotic substance, as in the case of certain genetic polymorphisms. Biomarkers of effect, also known as biological response biomarkers, are observable and measurable changes in an organism’s biochemical, molecular, cellular, or functional components resulting from exposure to chemical contaminants. These changes may be associated with disease development and provide valuable information about the body’s response to chemical compounds, thus aiding in human biomonitoring. Measured mainly in blood and urine samples, they can also indicate physiological or pathological biological processes at specific moments in the exposure–disease interaction [16].

Monitoring these biomarkers makes it possible to detect the effects of pesticide exposure before individuals experience adverse health effects, allowing for early intervention and prevention [8,14]. It also contributes to developing risk assessment strategies and implementing preventive measures to protect the health of people exposed to pesticides [4,17].

Currently, the best-known biomarkers of early effect are essentially associated with damage to the nervous system, changes and damage to DNA, and endocrine disorders [2,14,15,18,19,20]. Monitoring cholinesterase inhibition and related biomarkers can be used to assess pesticide exposure, so it is crucial to rapidly and comprehensively identify the biomarkers associated with pesticides in the body due to the multiplicity of the active substances they contain [2,8,13,14].

This will help us determine the level of risk to which workers and the general population are exposed. Therefore, one future trend associated with using biological monitoring to assess the risks of pesticide exposure is the development of new techniques to analyse multiple pesticides or mixtures of pesticides. Additionally, it is essential to increase the study of new biomarkers to ensure health protection and prevent irreparable damage to health [2].

Therefore, this systematic review aims to summarise innovative biomonitoring techniques for assessing pesticide exposure in agricultural workers, their advantages and limitations, and their applicability.

## 2. Materials and Methods

The *Preferred Reporting Items for Systematic Reviews and Meta-analyses* (PRISMA)^®^ methodology guidelines were followed to prepare this systematic review (Table S1) [21].

### 2.1. Eligibility Criteria

For search and selection, the eligibility criteria were that all articles reporting new techniques for biomonitoring pesticide exposure should be included. In addition, studies conducted on agricultural workers or in the context of agriculture were considered eligible. Articles dealing with common biomonitoring techniques or lacking innovation were excluded, as were literature reviews and conference papers. Only publications written in English were included.

### 2.2. Information Sources and Search Strategy

Four databases were used to search for articles: Medline/PubMed, ScienceDirect, Scopus, and Web of Science. The publication range considered for this study was from 2018 to 2023 to ensure a review of the most up-to-date results that meet the defined objective. The database search was conducted using the following keywords: [innovation OR “new techniques” OR “new technolog*”) AND (biomonitoring OR biomarkers OR “biological monitoring”) AND (“pesticide exposure” OR “pesticide *exposure”) AND (“agricultural workers” OR farmworkers OR farmer OR agriculture)]. The search of databases was conducted using the following criteria: ‘Article Title’, ‘Abstract’, and ‘Keywords’. Additionally, the references of the selected articles were analysed to include as many relevant articles as possible in line with the defined objective.

### 2.3. Selection Process

After entering the keywords, the databases were filtered by date (publications between 2018 and 2023), document type (articles), source type (journals), and language (English). Articles were excluded if they assessed common techniques for the biomonitoring of pesticides and metabolites, or biomonitoring in animals without validation studies in humans, or if the scope of the studies was outside the scope of the research.

All data from the search and selection of articles were recorded in an Excel spreadsheet. After the search, the articles were imported into the *Mendeley Reference Manager 2.107.0*^®^ software, and duplicates were eliminated.

The titles and abstracts were then analysed independently by two reviewers. Any disagreements between the reviewers were the subject of discussion and resolution between the reviewers. The selection of studies for the systematic review was based on a full and independent assessment of relevant articles and data collection. The process of selecting and excluding articles was documented in a separate document, accompanied by the relevant justifications.

### 2.4. Data Collection Process and Data Items

The following information was taken from the studies selected for inclusion: the innovative technique’s identification, the country where it was developed, the type of study conducted, the methodology used, information on the validation (or lack thereof) of the technique, its advantages and limitations, and the reference.

### 2.5. Risk of Bias Assessment

The *Cochrane Risk of Bias in Non-Randomized Studies of Interventions* (ROBINS) guidelines were used to analyse the risk of bias in the included studies [22].

## 3. Results

### 3.1. Study Selection

**Figure 1:**
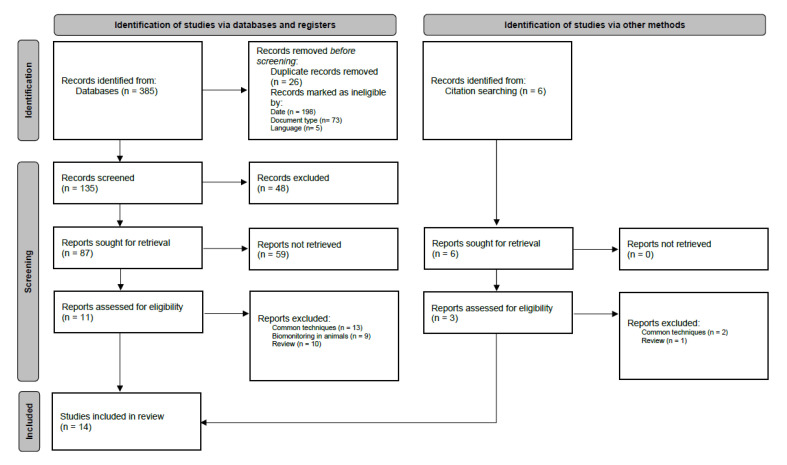
The PRISMA 2020 flow diagram for this systematic review.

A total of 14 articles were included in the systematic review. Figure 1 describes the process of article selection.

### 3.2. Included Studies’ Characteristics

The included studies had variable designs and objectives. Four were observational [23,24,25,26], three were cross-sectional [27,28,29], one was experimental [30], and six focused on developing and validating new biomonitoring methods [1,31,32,33,34,35]. Table 1 presents the general characteristics of the included studies, including author, year of publication, country of origin, study design, and participant information, and presents the innovative techniques that were developed and the methods used to validate them.

Regarding the location of the studies, 41.6% were conducted in Asia, 33.3% in Europe, and only 25% in America. Only one study was conducted on the African continent [28]. The study participants were agricultural workers who had been exposed to pesticides. Some of the participants were assigned to control groups and were not exposed to pesticides. The number of participants ranged from 3 to 1300. Based on the analysis of publication dates, it can be inferred that 2018 and 2019 saw the highest number of papers involving the development of new techniques in the field of biomonitoring exposure to pesticides.

### 3.3. New Methodologies and Techniques for Biomonitoring Pesticide Exposure

From 2018 to 2023, research on biomarkers associated with exposure to various pesticides has been conducted, although on a smaller scale than that linked to their use.

New methodologies and techniques for biomonitoring exposure to pesticides have identified several exposure and effect biomarkers, namely oxidised guanine [27], circulating cell-free DNA [23], IgG class autoantibodies [25], different dialkylphosphates [1,31,32], HSA-miR-199a-5p [26], Ethylene-thiourea [24], glutathione [28], malondialdehyde [28], 8HdG [28], BChE [29], AMPA-Fluazinam, DAPA-Fluazinam, 3,5,6-Trichloro-2-pyridinol, 6–Chloro Nicotinic Acid, and 3–Hydroxy–tetrahydrophthalimide [34].

Most of the biomarkers used in the included studies were associated with exposure to organophosphates [1,24,32,34,35]. These biomarkers were associated with modifications to the nervous system, neurological and endocrine problems, tumours, and genotoxic effects [18,25,36,37]. To detect and quantify participant biomarkers, biomonitoring studies mainly used urine [1,24,28,31,32,33,34] and blood samples [23,25,27,28,29,35].

### 3.4. Advantages and Limitations of New Methodologies and Techniques for Biomonitoring Pesticide Exposure

Most methodologies combined and/or improved traditional methods to overcome their limitations. This results in more sensitive, specific, and precise techniques with lower detection and quantification limits [1,28,29,31,32,34].

Table 2 presents a detailed compilation of the advantages and limitations of the new techniques found in the included studies, as reported by the authors, and the distinction of the biomarkers addressed as biomarkers of exposure or effect.

These studies have identified several advantages of the techniques, including high sensitivity and precision, fast application, simplification of inherent processes (such as chromatography extraction), and improved limits of detection and quantification of biomarkers [1,31,34].

The techniques have all been validated using various methods, resulting in effective biomonitoring techniques for pesticide exposure and complementary tools for assessing the associated risks. However, the studies revealed some limitations, including, in some cases, a small sample size that prevents the generalisation of the results, difficulties of practical application, a lack of consideration for genetic variations, and an inability to analyse the relationship between exposure to pesticides and the observed health effects.

### 3.5. Risk of Bias in Studies

Regarding the risk of bias in the studies, it was possible to verify that six studies have a low risk, seven have been classified as having a moderate risk of bias, and one has a high risk of bias. For this reason, it is important to analyse the results carefully.

## 4. Discussion

Innovative techniques in the field of pesticide biomonitoring have particularly been developed to improve existing methodologies and techniques by addressing and improving their limitations. For this reason, several studies have combined/improved liquid chromatography (LC) and gas chromatography (GC) methods to overcome the (individual) limitations associated with these techniques by other authors, namely inadequate recoveries and high coefficients of variation, as well as complex and time-consuming sample handling, which make their use in routine analysis unfeasible [1,28,31,32,34]. An immunochromatography test strip (ICTS) method was also validated using liquid chromatography to concurrently detect butyrylcholinesterase (BChE) activity and total BChE in human plasma samples after exposure to organophosphate insecticides. In the field of biomonitoring, this is a significant innovation. The method is considered innovative. It provides an affordable, quick, and accurate tool for assessing organophosphate exposure. It has potential utility for monitoring exposure in agricultural workers [35].

The satisfactory results obtained for linearity, recovery, precision, limit of detection, and limit of quantification, as well as the results obtained for variation compared with more complex methods, validate these new techniques and demonstrate their applicability in the context of biomonitoring for the assessment of exposure to pesticides in agricultural workers (and beyond), presenting them as facilitating and reliable tools for this assessment. However, the authors of these new or improved techniques point out that more studies using them are needed to validate their results more robustly, as in many cases, the characteristics of the sample used to carry out the validations are not ideal, and therefore, the data may not be generalisable or accurate [1,28,29,30,31,32,33,34].

Most studies found that the biomarkers for identification and quantification were related to organophosphate pesticides [1,24,28,30,31,32,33,34,35]. This may be because organophosphate pesticides are extensively applied in agriculture and are the pesticides most often mentioned by farmers when asked which products they use most [28]. Organophosphates are insecticides, and given the increasing appearance of pests on crops, they have emerged as a solution for their protection and associated income [1,24,28,30,31,32,33,34,35].

When analysing the biomarkers used, it is possible to associate them with serious health risks since their detection in the samples presupposes (often chronic) harmful exposure to agricultural workers [13,14,15,19]. The presence of biomarkers such as oxidised guanine is indicative of DNA damage likely caused by exposure to reactive oxygen species, which may be the result of environmental factors such as exposure to pesticides and is relevant for assessing the genotoxic effects of occupational exposure to pesticides [2,8,18,27].

Circulating cell-free DNA (ccfDNA) indicates cell damage and apoptosis, and its high levels have been associated with various pathological conditions, including cancer and non-malignant diseases. In the context of pesticide exposure, ccfDNA is being investigated as a potential biomarker of genotoxicity. However, further research is needed to validate this biomarker as a reliable biomarker of pesticide exposure, given the unsatisfactory outcome compared to the *Cytokinesis-block micronucleus cytome* assay in exposed individuals [23]. An increase in DNA damage and cell death was detected in the exposed group by another test, the *buccal micronucleus cytome* (BMCyt) assay [29]. The same study found that in silico analysis provided information on how metal ions could damage DNA and lead to apoptosis. The study also found that individuals with the XRCC1 Arg/Arg genotype may have some protection against genotoxic effects [29].

As a new technique for assessing exposure and damage to organophosphates, the potential use of autoantibodies as biomarkers of pesticide-induced neurotoxicity was presented. The study suggests that a significant increase in serum autoantibodies to several neuronal cytoskeletal proteins is associated with chronic exposure to this type of pesticide. However, it is necessary to conduct further research to determine whether these elevated levels of autoantibodies reflect a general neurogenic response or a specific pattern indicative of pesticide-induced neurotoxicity [25].

Dialkylphosphate metabolites are non-specific metabolites resulting from organophosphate metabolism, indicating nervous system changes and damage. They are usually measured in human urine due to their highly polar nature and low blood concentrations. Detecting these metabolites indicates recent exposure to this class of pesticides and allows for the determination of a possible correlation with health effects [1,31,32]. Coupling liquid chromatography to mass spectrometry for the detection of DAPs was found to be consistent among the studies that assessed exposure using this method and therefore appears to be an improved liquid chromatography method (the most widely used method for the determination of organophosphates in urine samples) that can be used for pesticide risk assessment [1,31,32].

The microRNA HSA-Mir-199a-5p is a biomarker that can indicate a biological response to chronic pesticide exposure in agricultural workers. It is commonly used in clinical studies to assess the effects of chemical exposure or the progression of diseases. This biomarker has a good predictive value for exposure to harmful pesticides, which could be crucial for monitoring strategies and potentially for prematurely detecting diseases associated with pesticide exposure. Although the results are promising, the study acknowledges the necessity for further validation with a larger sample size and the evaluation of additional miRNAs to strengthen the findings [26].

FRET-based nanosensors represent a significant advancement in detecting cellular responses to environmental chemicals, specifically pesticides. They have been shown to detect and classify phenotypic changes in macrophages caused by very low concentrations of various pesticides, which are lower than the detection limits of traditional toxicological methods. The array-based sensor is a sensitive and high-performance tool that can potentially improve the safety of chemical use in the environment. It enables the early detection of subtle cellular changes that may indicate chronic toxicity [30].

Mandić-Rajčević and Colosio (2019) conclude that accurate estimation of skin exposure in pesticide workers is essential for risk assessment and the assessment of absorbed doses in workers. Their study states that the patch method is useful, but it requires a method of outlier finding to detect extreme values that could indicate an overestimation of exposure. A significant number of extreme values were effectively identified by the modified Z score, which were then processed to refine the accuracy of the skin exposure estimates. Biomonitoring results supported the validation of this method. The correlation between estimated dermal exposure to mancozeb and the determined levels of ETU (a principal metabolite of mancozeb) was better. The article recommends improving the exposure and risk assessment method for pesticide use in agriculture; this or other appropriate methods should be used to detect discrepant values [24].

A study has validated the Predicted Exposure Assessment Model (WHO-PEAM) developed by the *World Health Organization* and concluded that it is a reliable tool for estimating exposure to chlorpyrifos. The model demonstrated strong agreement with biological monitoring data, and its predictions were comparable to the daily absorbed doses derived from biological monitoring. This suggests that there is no significant difference between the two methods. The study suggests that the WHO-PEAM model can be a valuable screening tool for managing health risks in farmers exposed to pesticides. It also highlights the potential for adapting the WHO-PEAM model to other conditions. However, further external validation in different farmer populations is necessary to ensure the reproducibility of exposure dose estimates [33].

This review has some limitations that need to be addressed to ensure the most accurate data analysis. One of the limitations is that the innovative techniques largely relate to organophosphate biomarkers and more studies are needed for other groups of pesticides. Another limitation is related to the risk of bias present in some of the included studies, requiring greater caution when analysing the results associated with them.

New biomonitoring methods for assessing pesticide exposure must be fully analysed and tested. The benefits of these techniques can complement qualitative risk assessments and help develop strategies to protect public health and the environment.

## 5. Conclusions

The emergence of new techniques for biomonitoring pesticide exposure is associated with the improvement in traditional techniques and their combination with more specific techniques that facilitate the assessment of exposure to these products in agricultural workers and beyond.

These new techniques have advantages such as greater sensitivity and precision, less or non-invasive techniques, faster application, and improved associated processes. However, they also have limitations that need to be improved to obtain the most accurate and realistic results.

The authors suggest that these limitations could be addressed by increasing the number of studies based on these techniques to assess the methods’ repeatability, effectiveness, and practicality. In addition, several authors report exposure to pesticides that are highly toxic to health and the environment in qualitative assessments of agricultural workers’ pesticide exposure.

Under these conditions, biomonitoring studies must be carried out to obtain a quantitative assessment of exposure, which will facilitate the adoption of measures to protect the health of workers and allow us to gain a better understanding of the health effects of exposure to different active substances that are still unknown and/or underestimated.

## Figures and Tables

**Table 1:** Summarised information about the methodology of the included studies.

Author(s)	Country	Study Type	Methodology	Validation
[27]	Brazil	*Cross-sectional* Farmworkers exposed (N = 137) C (N = 83)	Alkaline comet assay modified with limitation enzyme (hOGG1: human 8-oxoguanine DNA glycosylase) to identify **oxidised guanine**.	*Comparison with buccal micronucleus cytome assay, global methylation, haematological parameters,* *biochemical analyses, and particle-induced X-ray emission (PIXE) method.*
[23]	Turkey	*Observational cohort* Greenhouse workers (N = 72) C (N = 51)	Cytokinesis-block micronucleus assay (CBMN) + measurement of circulating **cell-free DNA** (ccf-DNA) in the blood of pesticide-exposed greenhouse workers using a Qubit^®^ fluorometer to assess DNA damage in lymphocytes.	*Tests with high-sensitivity kits, Quant-it™.*
[25]	Egypt	*Observational cohort* Greenhouse workers (N = 72) CG (N = 51)	*Western blot* analysis to verify the presence of **IgG class autoantibodies** against ten specific neuronal and glial proteins (indicators of brain damage).	*Neutralisation of each autoantibody in the serum with its target protein; unpaired *t*-test*
[1]	Spain	*Validation Method* N = 20	*Liquid chromatography–tandem mass spectrometry (LC-MS/MS)* for quantitative determination of six **dialkyl phosphates (DAPs)** in human urine samples + liquid extraction (LLE) method with a salting-out procedure (without pre-treatment or purification).	*Linearity, accuracy, precision, quantification limits, and specificity.* *High recoveries and accurate results.*
[26]	Italy	*Observational case-control* Farmworkers (N = 28) C (N = 9)	Analysis of levels of **HSA-miR-199a-5p** by droplet digital PCR (ddPCR) on liquid biopsy samples + absolute quantification of levels with QuantaSoft software + statistical analyses.	*Receiver operating characteristic (ROC) curves.*
[30]	*Not specified*	*Experimental*	Robust detection of **macrophage responses** to femtomolar concentrations of common pesticides (orders of magnitude lower than traditional ones) using specific *Förster resonance energy transfer* (FRET) sensors.	*Sensitivity and specificity.* *Linear Discriminant Analysis (LDA).*
[31]	India	*Validation Method* Individuals exposed during cultivation (N = 100) C(N = 50)	A method linking *ultrafast liquid chromatography with tandem mass spectrometry (UFLC–MS/MS)* for the detection of **dialkyl phosphates (DAPs)** in human urine samples.	*Selectivity, linearity, limit of detection and quantification, recovery, precision and accuracy, matrix effect, and stability.*
[24]	Italy	*Observational* Agricultural workers (N = 27)	Estimative dermal exposure with a patch methodology + 24 h post-exposure urine biomonitoring for ***Ethylene-thiourea* (ETU)** + detection of outliers via multiplication of the median, the median absolute deviation, and boxplots.	*Modified Z score.*
[28]	Morocco	*Cross-sectional and prospective cohort* Farmworkers (N = 300) Pregnant women’s (N = 1000)	Multiple sample analysis by LC-MS and GC-MS analytical methods for biomonitoring pesticide metabolites with oxidative stress biomarkers (**Glutathione, Malondialdehyde, and 8-OHdG**).	*Calibration curves comparable to the matrix for each analytical procedure, establishing basal concentrations of pesticides with samples from participants without exposure.*
[29]	Brazil	*Cross-sectional observational* Farmworkers (N = 76) C (N = 72)	*Particle-induced X-ray emission* (PIXE) technique for the identification and quantification of inorganic elements in blood samples + **buccal micronucleus cytome assay** (BMCyt) (genotoxicity biomarker) + in silico analysis.	*Standard protocols, Mann–Whitney test, Spearman’s correlation + Bonferroni correction*
[32]	China	*Validation Method* Farmworkers (N = 3) C (N = 3)	Vortex-assisted salt-induced liquid–liquid microextraction (VA-SI-LLME) + liquid chromatography/tandem mass spectrometry (UHPLC/MS/MS) for the biomonitoring of **organophosphate pesticide metabolites** in human urine samples.	*Linearity, recovery, precision, limit of detection (LOD), and limit of quantification (LOQ).*
[33]	Vietnam	*Validation Method* Farmworkers (N = 18)	Application of a questionnaire to obtain parameters for predicting daily doses of **chlorpyrifos exposure** without biological monitoring + comparison with absorbed daily dose (ADD) measured by biomonitoring.	*Wilcoxon Test.*
[34]	Italy	*Method development*	QueChERS procedure + *UHPLC-MS/MS* + *GC-MS/MS* method for ***Fluazinam, Chlorpyrifos, Ethyl, Methyl, Buprofezin, Boscalid, Imidacloprid, Thiacloprid, Phosmet, Captan, and Metiram*** quantification.	*Linearity, recovery, and analysis of variance (one-way ANOVA).*
[35]	United States, Pakistan	*Validation Method* Farmworkers (N = 124)	Immunochromatographic test strip (ICTS) for the simultaneous detection of **BChE** activity and **total BChE** in human plasma samples exposed to organophosphorus pesticides.	*Comparison of results between immunochromatographic test strip (ICTS), liquid chromatography–tandem mass spectrometry (LC-MS/MS) (standard and highly accurate analytical technique), and Pearson’s correlation.*

**Note:** C = control group; N = number of participants.

**Table 2:** Advantages and limitations of new techniques identified in included studies.

New Technique	Biomarkers	Advantages	Limitations	Reference
*Alkaline Comet Assay Modified with limitation enzyme (hOGG1: human 8-oxoguanine DNA glycosylase)*	Effect biomarker	Sensitive and specific detection of oxidised guanine; Assessment of DNA damage at the molecular level; Method combined with non-invasive and precise techniques.	The test does not provide information on cell repair or the persistence of DNA damage; Failure to detect variations in damage; The study design does not allow links to be established between pesticide exposure and observed health effects.	[27]
*The potential of ccf-DNA as a biomarker for pesticide exposure*	Exposure biomarker	A less invasive technique capable of detecting cellular processes in real time; A stable and adequate biomarker for pesticide biomonitoring; Quick and easy measurement with accurate equipment.	Limitations in the interpretation of ccf-DNA as the only indicator of genotoxicity. Sample characteristics and generalization; Confounding factors are not considered.	[23]
*The detection of serum autoantibodies against neural proteins as a biomarker for pesticide-induced neurotoxicity*	Effect biomarker	A non-invasive technique; Serum autoantibodies as biomarkers; High specificity and direct association with brain lesions; The use of Western blot analysis as a standard measure.	Sample characteristics and generalization; More related studies are needed to support the obtained results.	[25]
*The development of a new method to measure dialkyl phosphates in human urine using liquid chromatography–tandem mass spectrometry*	Exposure biomarker	Simpler and faster analysis than gas chromatography; Optimum chromatographic determination in a quicker time; A method validated according to ISO/IEC 17025; Simplification of the extraction process; Accurate limits of quantification (LOQs); Suitable for routine urine analysis in biomonitoring.	The presence of a significant matrix effect (ME) in the determination of urinary DAP; Availability and the cost of individual isotopically labelled internal standards for each DAP; A calibration curve for synthetic urine.	[1]
*The use of a high-sensitive droplet digital PCR assay to identify changes in the levels of the selected microRNA*	Exposure biomarker	High sensitivity and accuracy in the absolute quantification of nucleic acids, ideal for the monitoring of epigenetic changes associated with exposure to pesticides; Robust against inhibitors commonly found in biological samples, allowing for reliable analysis in complex matrices such as serum and plasma.	Sample size; Possible involvement of other miRNAs or complex interactions between multiple epigenetic factors; Not approved by the ethics committee; Pilot study.	[26]
*A FRET-based nanosensor array planned to extend responses to modifications in cell surfaces regarding pesticide exposure*	Effect biomarker	High sensitivity and lower detection limits than current cell-based methods; A sensor matrix with differentiated reactions to diverse groups of pesticides.	Dependence of macrophage cell lineage models; The specificity of the sensor set for different types of pesticides; Difficulties in practical application.	[30]
*A new UFLC–MS/MS method for the detection of organophosphate pesticide metabolites in urine*	Exposure biomarker	A sensitive, accurate, and selective method; A small sample volume; Efficiency in extracting target analytes; Minimal matrix effect; Maintains analyte stability.	*Not specified*	[31]
*Methods for identifying and validating outliers in biological monitoring*	Exposure biomarker	Coverage of the patch methodology; Modified Z score accuracy; The use of a previously validated data collection form; A significant sample volume; A standardised methodology for application in future risk assessment studies.	Sample characteristics; A lack of “real” exposure measurement; Data variability; Limitations of the adhesive method in terms of dermal exposure.	[24]
*The integrated use of human biomonitoring of exposure:* The *PaPOE study*	Exposure and effect biomarkers	A comprehensive approach to assessing the impact of pesticide exposure on the epigenome; The study design allows for an analysis of the immediate and long-term epigenetic effects of exposure to pesticides; The integration of biomonitoring techniques; A contribution to policymaking.	Sample characteristics and generalization; Genetic variation is not considered; Understanding the isolated effects of pesticide mixtures can be challenging due to their complexity; A lack of couples in the sample to determine the influence of each occupational exposure on the offspring; A loss to follow-up of participants; The results are not shared individually.	[28]
*Advanced molecular techniques and bioinformatics tools (particle-induced X-ray emission (PIXE)) for the detection and quantification of inorganic elements in blood samples*	Effect biomarker	Comprehensive use of BmCyT; Quantitative detection of inorganic elements in the blood using PIXE as a measure of exposure to harmful substances present in pesticides; The inclusion of polymorphic genetic analyses (XRCC1 and PON1 genotypes) relevant to DNA repair and pesticide metabolism (respectively); The use of in silico analysis to model potential interactions.	Failure to analyse causal inferences between pesticide exposure and observed genotoxic effects; Sample characteristics and generalization; Limited sensitivity for a butyrylcholinesterase (BChE) biomarker in the study; Genetic variation.	[29]
*VA-SI-LLME-UHPLC/MS/MS*	Exposure biomarker	A short agitation time and fast extraction (six minutes in total); High sensitivity and accuracy; Profitable and environmentally conscious (use of only organic solvents); Efficiency in practical applications.	*Not specified*	[32]
*Validation of the World Health Organization Predicted Exposure Assessment Model (WHO-PEAM) for chlorpyrifos exposure*	Exposure biomarker	Exposure prediction without biomonitoring; A more accessible technique; A consistent alternative for assessing exposure to chlorpyrifos given the correlation coefficient values; A practical tool for risk management.	The influence of the sample size on statistics; The total variability is not considered; The study did not include activities that could increase exposure, such as circulation in recently treated areas; The need to improve the parameters addressed to increase accuracy.	[33]
*A multi-analyte method using LC-MS/MS and GC-MS/MS for the quantification of pesticides and metabolites in human urine*	Exposure biomarker	An accurate, consistent, easy, cheap, and fast method; Extensive applications in biomonitoring; A multi-analyte approach; Satisfactory results for linearity, recovery, and accuracy.	The PSA adsorbent is inadequate; Unacceptable results for linearity, accuracy, and precision regarding 3-OH THPI; The need for sophisticated analytical equipment and complex sample preparation; Dermal and inhalation exposure routes are not considered.	[34]
*Innovative sandwich immunoassay-based immunochromatographic test strips (ICTSs) for measuring butyrylcholinesterase (BChE) activity and the total amount of BChE in human plasma samples*	Exposure biomarker	Simultaneous measurement of BChE activity values from a small sample volume; The use of a monoclonal antibody simplifies the assay, avoiding the need to use multiple antibodies; A rapid approach; A cheap and portable method.	*Not specified*	[35]

## Data Availability

The raw data supporting the conclusions of this article will be made available by the authors upon request.

## Supplementary Materials

The following supporting information can be downloaded at https://www.mdpi.com/article/10.3390/toxics13020104/s1: Table S1: PRISMA 2020 Checklist.

## Abbreviations

The following abbreviations are used in this manuscript:

PRISMA	*Preferred Reporting Items for Systematic Reviews and Meta-analyses*
ROBINS	*The Cochrane Risk of Bias in Non-Randomized Studies of Interventions*
DNA	Deoxyribonucleic acid
IgG	Linear dichroism
LC-MS/MS	Liquid chromatography–tandem mass spectrometry
DAPs	Dialkyl phosphates
CBMN	Cytokinesis-block micronucleus assay
hOGG1	Human 8-oxoguanine DNA glycosylase
ccf-DNA	Circulating cell-free DNA
LLE	Liquid extraction
ddPCR	Droplet digital PCR
FRET	Förster resonance energy transfer
ETU	Ethylene-thiourea
GC-MS	Gas chromatography–mass spectrometry
VA-SI-LLME	Vortex-assisted salt-induced liquid–liquid microextraction
UHPLC/MS/MS	Ultra-high-performance liquid chromatography
ADD	Absorbed daily dose
QueChERS	Quick, Easy, Cheap, Effective, Rugged, and Safe
ICTS	Immunochromatographic test strip
BChE	Butyrylcholinesterase
LOD	Limit of detection
LOQ	Limit of quantification
ROC	Receiver operating characteristic
PIXE	Particle-induced X-ray emission
BMCyt	Buccal micronucleus cytome assay
WHO-PEAM	Predicted Exposure Assessment Model by World Health Organization
